# Effects of meteorological factors on the retention of particulate matter in lawn grass blades

**DOI:** 10.3389/fpls.2025.1495212

**Published:** 2025-01-22

**Authors:** Junrui Wang, Weihan Kong, Haimei Li, Xiaodan Sun, Yingkun Sun, Yu Liu

**Affiliations:** ^1^ College of Landscape Architecture and Forestry, Qingdao Agricultural University, Qingdao, Shandong, China; ^2^ Marine Ecology Research Center, First Institute of Oceanography, Ministry of Natural Resources, Qingdao, China

**Keywords:** turfgrass, particulate matter, dust retention, rainfall, extreme wind speed

## Abstract

Plant leaves can reduce the concentration of atmospheric particulate matter (PM) by absorbing it in the air, and this mitigates the deleterious human health effects of PM. However, the ability of plant leaves to retain dust is limited and varies continually due to various meteorological factors such as rainfall, extreme wind speed, and PM_10_ concentrations. Here, we measured the ability of seven types of turfgrass with leaves similar in macromorphology but varying in micromorphology to retain dust particles of different sizes; we also analyzed the effects of various meteorological factors, such as rainfall, maximum wind speed, and PM_10_ concentration, on the ability of leaves to retain particles of different sizes. There were significant differences in the ability of the seven types of turfgrass to retain particles of different sizes; the dust retention capacity of Zoysia sinensis was the strongest(2.04 g·m^-2^), and that of *Festuca elata* was the weakest(1.39 g·m^-2^). The elution rates of PM>10 after rainfall of 3 mm and 4 mm were significantly higher than those of PM_2.5-10_ and PM_2.5_; the elution rates of PM_>10_, PM_2.5-10_, and PM_2.5_ increased as the amount of rainfall increased. When the amount of dust on leaves is low, wind promotes increases in leaf PM retention. When the blade retains a certain amount of dust, the maximum wind speed is greater than 9.1 m·s^-1^, which leads to a decrease in the dust retention of lawn grass blades. The concentrations of PM_10_ and PM_2.5_ were positively correlated with the retention of particles of different particle sizes. Therefore, evaluations of the dust retention ability of plant leaves require consideration of the effects of local rainfall, maximum wind speed, PM_10_ concentration, and other factors.

## 1 Introduction

The acceleration of urbanization has led to a rapid decline in urban air quality, and air pollution caused by atmospheric particulate matter (PM) has become an issue of wide concern (Zhou et al., 2021). TSP (Total Suspended Particulates) stands for particles that can be suspended in the air and have an aerodynamic equivalent diameter of less than 100 microns, that is, particles with a particle size under 100 microns.These particles can enter the body during breathing and pose a health hazard. Suspended in the atmosphere for a long time without settling, reducing the atmospheric visibility; Participate in atmospheric chemical reactions, increase the degree of pollution (Huang et al., 2012). Particles with an aerodynamic diameter of less than 2.5 μm (PM_2.5_) can pose more harm to human health compared with particles of other sizes because fine particles may penetrate the lining of the alveolar ducts, which promotes the entry of toxins into the bloodstream (Kwon et al., 2020; Qin et al., 2021; Rodriguez-Germade et al., 2014) and increases the incidence of malignant tumors (Karottki et al., 2015; Nowak et al., 2018; Qin et al., 2019). Previous studies have shown that urban plants can effectively retain PM in the atmosphere and alleviate urban PM pollution (Go et al., 2021; Popek et al., 2018; Vos et al., 2013). Urban plants can reduce the production of surface PM (Escobedo et al., 2011) and reduce the concentration of atmospheric PM by absorbing airborne PM on the surface of their leaves. Precipitation of PM is promoted by improving micrometeorological conditions (Beckett et al., 1998; Freer-Smith et al., 2005; Hofman et al., 2014).

The ability to retain PM on the leaf surface varies among species (Bui et al., 2021; Marien et al., 2019; Xu et al., 2018; Zhang et al., 2020), and this is associated with interspecific variation in leaf morphology (Leonard et al., 2016). Macromorphological leaf characteristics include traits such as leaf height, leaf size, and leaf whorl arrangement (Leonard et al., 2016; Nowak et al., 2006; Rasanen et al., 2013). Leaf micromorphological characteristics include traits such as villous number (Burkhardt, 2010; Chiam et al., 2019), surface roughness (Hwang et al., 2011; Sgrigna et al., 2020), groove ratio (Chen et al., 2017; Liang et al., 2016), and wax layer content (Perini et al., 2017; Saebo et al., 2012). Interactions among these leaf structural characteristics significantly affect the retention of PM in plant leaves (Guerrero-Leiva et al., 2016; Leonard et al., 2016; Li et al., 2020; Sun et al., 2018). Previous studies have analyzed the dust retention capacity of substances by sampling leaves after long periods of no precipitation or at the end of the growing season (Bui et al., 2021; Guerrero-Leiva et al., 2016; Xu et al., 2018); however, the amount of dust retained by urban plants in urban environments varies with meteorological factors, such as rainfall, wind, and dust concentrations (Rodriguez-Germade et al., 2014). Previous studies have shown that particles retained on the leaf surface of trees can be removed by the cleaning effect of rainfall (Przybysz et al., 2014; Rodriguez-Germade et al., 2014; Wang et al., 2015), and these particles fall on the soil or ground with rain, where the organic components of PM can be decomposed by natural processes; the inorganic components of PM are fixed in the soil (Dzierzanowski et al., 2011). Coarse PM (particle size greater than 10 μm) in foliar particles is easily displaced from the leaf surface by rain, and approximately 30–41% of PM on the leaves of Pinus sylvestris can be removed by 20 mm of rainfall (Przybysz et al., 2014).

Steubing examined the erosion of pollutants on the surface of ryegrass and found that the amount of pollutants displaced significantly increased after 15 min of rainfall (Steubing, 1982). Rodriguez-Germade et al. demonstrated that rainfall washed away part of the PM accumulated on the leaves of Platamus hispamica (Rodriguez-Germade et al., 2014). Wang et al. found that rainfall events can cause a large loss of PM on Ligustrum lucidum leaves. Cumulative precipitation of 10.4 mm and 31.9 mm washed away 28% and 48% of PM in L. lucidum leaves, respectively (Wang et al., 2015). Xu et al. found that 51–70% of the particles on the blade surface were washed away within the experimental intensity range (Xu et al., 2017).

Wind speed is also an important factor affecting the amount of dust retained on urban plants, and plants are affected by wind when its velocity exceeds a certain threshold. Ould-Dada & Baghini, 2001 found that the amount of PM adsorbed on the leaf surface was not affected by wind when it was less than 5 m·s^-1^. Tiwary et al. (2006) found that wind affected the amount of dust retained on hawthorn, Buxus, and purple only when it exceeded 0.8, 1.2, and 1.7 m·s^-1^, respectively (Tiwary et al., 2006). The effects of meteorological conditions on particle retention vary (Weerakkody et al., 2018b), and these meteorological conditions continuously limit the PM removal capacity of plants (Chen et al., 2021).

Here, the particle retention abilities of seven common turfgrass species with similar macromorphological characteristics but different micromorphological characteristics was studied. We believe that different weather conditions and different surface micromorphology of the blade have great influence on its dust retention ability. Therefore, We used an *in-situ* sampling method to characterize changes in the dust retention of seven types of common lawn grasses and the influence of weather conditions and leaf surface micromorphology on leaf PM retention was discussed. Overall, our findings provide new insights into the mechanism of leaf PM retention and will aid future quantitative evaluations of the dust retention abilities of leaves.

## 2 Materials and methods

### 2.1 Plant materials

We studied the dust retention of the leaves of seven turfgrass species in Qingdao, China: *Liriope spicata*, *Lolium perenne*, *Festuca elata*, *Poa pratensis*, *Zoysia sinica*, *Cynodon dactylon*, and *Agrostis stolonifera* plants were obtained from the campus of Qingdao Agricultural University and transplanted into pots before the experiment. The rest of the experimental plants were planted in pots (length 40 cm, width 20 cm, height 15 cm) in mid-July 2022, with 8 pots for each plant. Experiments were performed after a lawn was formed. Before testing, the leaves were fully washed, cleaned, and left to dry for a day; they were then placed on the roadside for study of their dust retention ability. The morphological characteristics of the seven types of turf grasses are shown in **Table 1**.

**Table 1:** The morphological characteristics of the seven turfgrass species.

Plant	Family name	Morphological features
*Liriope spicata*	Liliaceae Juss	Leaf blade apex acute or obtuse, with 5 veins, midvein conspicuous, margin serrate.
*Lolium perenne*	Poaceae Barnhart	Leaf blade linear, soft, sometimes auriculae.
*Festuca elata*	Poaceae Barnhart	Leaf blade linear-lanceolate, apex long acuminate, often flattened, glabrous below, glabrous upper mask.
*Poa pratensis*	Poaceae Barnhart	Leaf blade linear, flattened or inrolled, apex acuminate, smooth or margin slightly coarse.
*Zoysia sinica* Hance	Poaceae Barnhart	Leaf blade light green or gray-green, abaxially pale, glabrous, slightly hard in texture, flat or curled in margin.
*Cynodon dactylon*	Poaceae Barnhart	Leaf blade linear, usually glabrous on both surfaces.
*Agrostis stolonifera*	Poaceae Barnhart	Leaf blade narrowly lanceolate, both surfaces rough, apex acute.

### 2.2 Sample collection

From September 2 to November 13, 2022, plant materials were sampled every three days at Chengyang Campus of Qingdao Agricultural University (36° 19’ 5” N, 120° 23 ‘52 “E). In the event of rainfall, plant materials were sampled one day before and one day after rainfall, and six replicate samples were taken for each species. During sample collection, the grass in a well-grown area of 10×10 cm was cut 1 cm away from the roots, placed in a labeled plastic bag to minimize shaking, and transported to the laboratory for testing.All plants used in this study had sufficient number of leaves for taking leaf samples during the experiment, and leaves newly produced during the experiment were not sampled.

The dust retention per unit leaf area was measured using the filtration mass method. The leaf sample was placed into a beaker and immersed in distilled water for 2 h; the attachment point on the leaf was cleaned with a brush, and the leaf was removed with tweezers. The leaf surface was rinsed again with a small amount of distilled water. The leaching solution was filtered using a microporous filter membrane with a diameter of 10 μm that had been dried and weighed, and the filter solution was filtered using a microporous filter membrane with a diameter of 2.5 μm that had been dried and weighed. Finally, the filtrate was poured into a beaker that had been previously weighed, and the beaker was placed in a 60°C oven and dried to a constant weight (i.e., the difference in two measurements was less than 0.0002 g). Next, the filter membrane of trapped particles was also placed in the oven at 65°C and dried to a constant weight, and the different particle sizes were classified as follows: particle mass > 10 μm (referred to as PM_>10_), 10–2.5 μm (referred to as PM_2.5–10_), and ≤ 2.5 μm (referred to as PM_2.5_). The sum of the three was referred to as TSP. The dried leaves were placed on a scanner (CanoScan 5600F) for scanning, and the obtained digital images were imported into ImageJ to calculate the leaf area S (m^2^); the dust absorption of the unit leaf area of the plant leaves was calculated using the following formula:


Q=(Q2−Q1)×S−1

where Q is the dust retention amount per unit leaf area (g ·m^-2^), Q2 is the mass of the filter membrane after filtration (g), Q1 is the mass of the filter membrane before filtration (g), and S is the leaf area of the sampled leaf (m^2^).

Variation in dust retention per unit leaf area was calculated using the following formula:


Dynamic variation M=M2−M1

where M2 represents the maximum dust retention per unit leaf area (g·m^-2^) and M1 represents the minimum dust retention per unit leaf area (g·m^-2^) during the sampling period.

The precipitation elution rate of particles with different particle sizes on the leaf surface was calculated using the following formula:


W=(P2−P1)×P2−1

where P2 represents the amount of dust retention per unit leaf area before rainfall (g·m^-2^), and P1 represents the amount of dust retention per unit leaf area after rainfall (g·m^-2^).

After excluding the effect of rainfall on foliar PM following previous studies (Beckett et al., 2010; Freer-Smith et al., 2004), data with maximum wind speed greater than 9 m·s-1 during the sampling period were used to study the effect of maximum wind speed on particle retention.

Variation in particle retention associated with the effect of maximum wind speed was calculated using the following formula:


M3=M5−M4

where M4 represents the dust retention per unit leaf area before the maximum wind speed (g·m^-2^), and M5 represents the dust retention per unit leaf area after the maximum wind speed (g·m^-2^).

### 2.3 Determination of leaf surface structure

**Figure 1:**
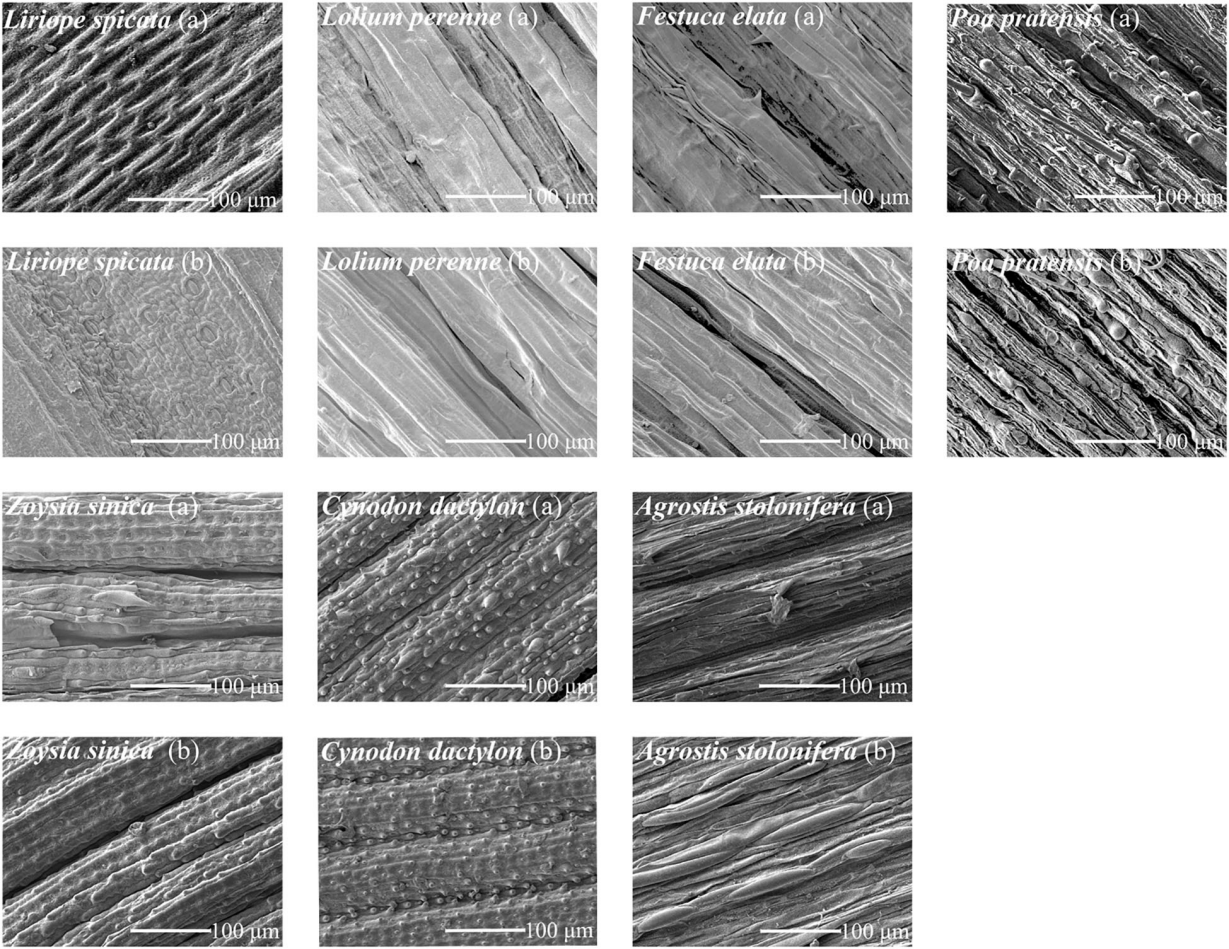
Scans of the surface of different turfgrasses. upper **(A)** and lower **(B)** leaf surface.

**Figure 2:**
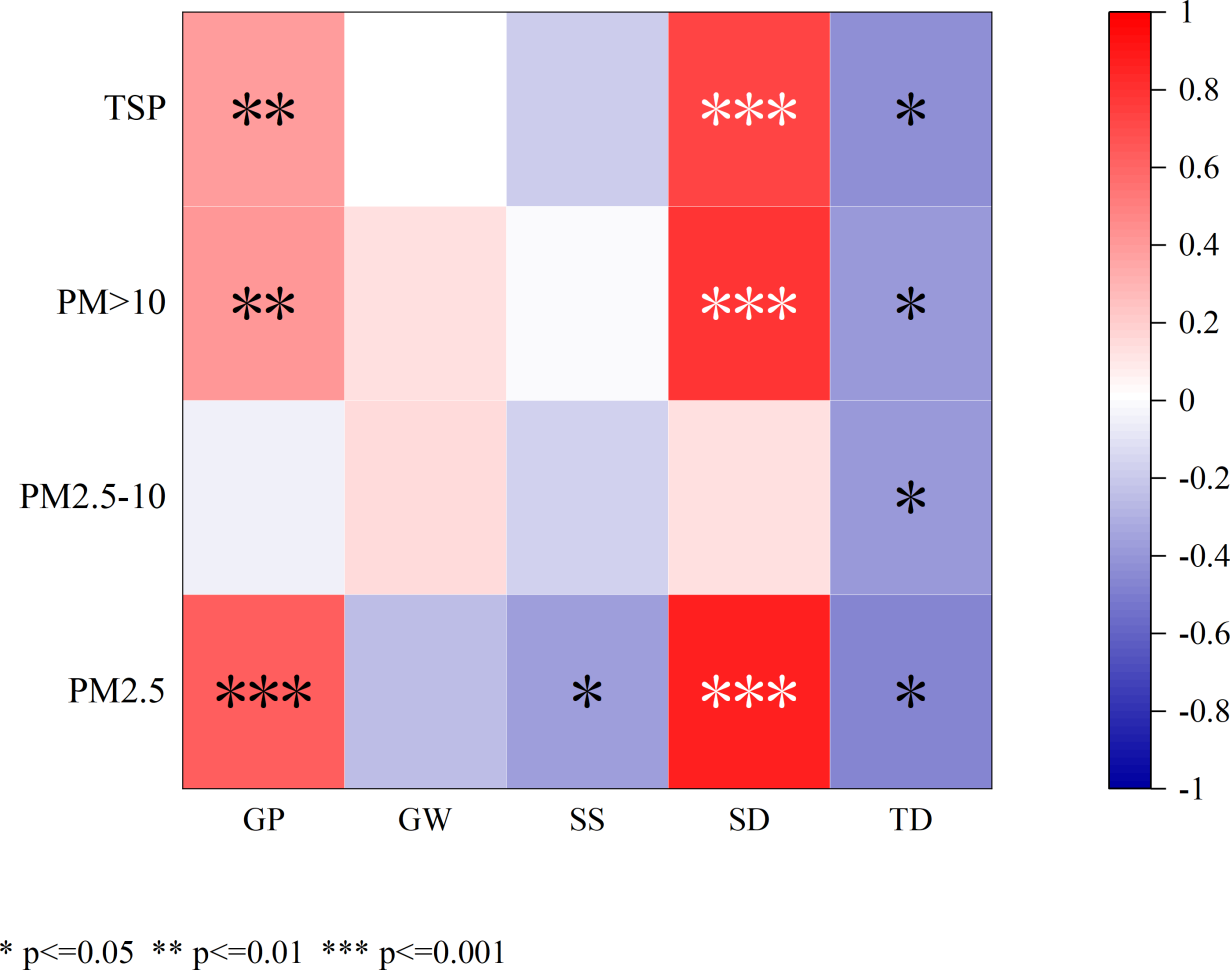
Correlations of the groove proportion, groove width, stomatal size, stomatal density, and trichoid density with the retention of TSP, PM>10, PM10, and PM2.5 per unit leaf area. "*" indicates (P < 0.05), “**” indicates P < 0.01.

Leaves were cut and placed in a clean plastic bag; care was taken to ensure that leaf villous were not damaged. A 3 mm × 3 mm tissue block was placed in the middle of the blade with a new blade; it was then placed into a small glass bottle and fixed in FAA(Formalin-Aceto-Alcohol) fixing solution for more than 4 h. After drying the sample using a vacuum, the samples were dehydrated using a graded ethanol series (60%, 70%, 80%, 90%, and 100% ethanol solution) for 10 min at each ethanol concentration and finally replaced with tert-butanol. The samples were then placed into a freeze-dryer for vacuum drying. After completely drying, the samples were removed and placed on the platform. A scanning electron microscope JSM-7500F (JEOL Ltd., Tokyo, Japan) was used to observe the surface structure of plant leaves at different magnifications (**Figure 1**). The surface microstructure parameters of 7 species of turfgrass were calculated (**Table 2**), and the correlation between particle retention per unit leaf area and that of turfgrass was analyzed (**Figure 2**).

**Table 2:** Microstructural parameters of the blade surface.

Species	GP (%)	GW (μm)	SS (μm^2^)	SD (N·mm^-2^)	TD (N·mm^-2^)
*L. spicata*	57±4a	28.89±6.11cd	225.83±23.42c	229.79±12.95a	–
*L. perenne*	34±3g	71.83±3.43a	364.77±70.14b	88.16±27.56c	–
*F. elata*	47±2bc	76.03±22.94a	490.05±31.97a	131.93±24.91b	45.26±6.50abc
*P. pratensis*	40±3de	47.75±11.62b	264.37±10.84c	154.88±16.11b	49.18±4.10ab
*Z. sinica*	50±9b	48.99±3.79b	135.15±11.14d	249.27±29.96a	35.57±6.17d
*C. dactylon*	38±2ef	23.72±0.83d	138.34±17.98d	141.56±6.30b	55.50±15.56a
*A. stolonifera*	44±3cd	40.44±7.12bc	161.37±38.63d	63.74±10.15d	42.27±7.85bc

Leaf surface microstructure parameters are expressed as mean ±SE, n=6. “GP” stands for “Groove proportion,” “GW” stands for “Groove width,” “SS” stands for “Stomatal size,” “SD” stands for “Stomatal density,” “TD” stands for “Trichome density,” and “-” stands for “No data”.

### 2.4 Collection of meteorological data

**Figure 3:**
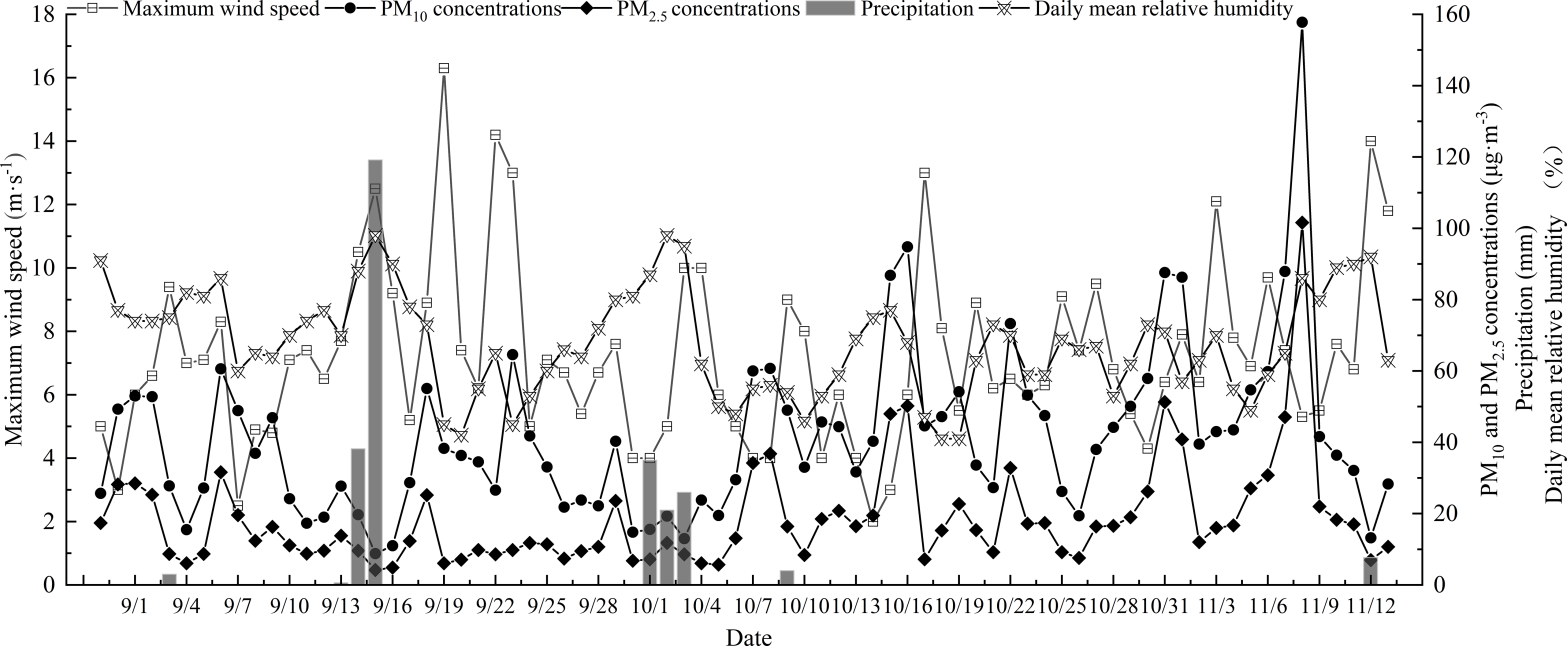
Diurnal variation in precipitation, maximum wind speed, PM10, and mean relative humidity.

Precipitation, relative humidity, and maximum wind speed data during the study period were obtained from the China Meteorological Data Network (http://data.cma.cn/). Data on PM_10_ and PM_2.5_ concentrations during the study period were obtained from the China Air Quality Online Monitoring and Analysis platform (https://www.aqistudy.cn/) (**Figure 3**). After excluding the effect of rainfall and maximum wind speed on the dust retention of turfgrass, correlations of the average daily PM_10_ concentration, average daily PM_2.5_ concentration, and average daily relative humidity at each sampling interval with the retention of particles of different sizes by the seven turfgrass species were analyzed.

### 2.5 Data analysis

Differences in the amount of particles (TSP, PM_10_, PM_2.5-10_, and PM_2.5_) retained on the leaf surface of seven types of turfgrass during the sampling period were analyzed by one-way ANOVA, and multiple comparisons were made using least significant difference (LSD) tests and Duncan multiple-range tests. All statistical analyses were performed using SPSS25.0 (SPSS, IBM, USA) software, and the threshold for statistical significance in all analyses was P < 0.05 (Chen et al., 2024).

## 3 Results

### 3.1 Comparison of the maximum retention capacity of particles of different sizes of lawn grass blades

**Figure 4:**
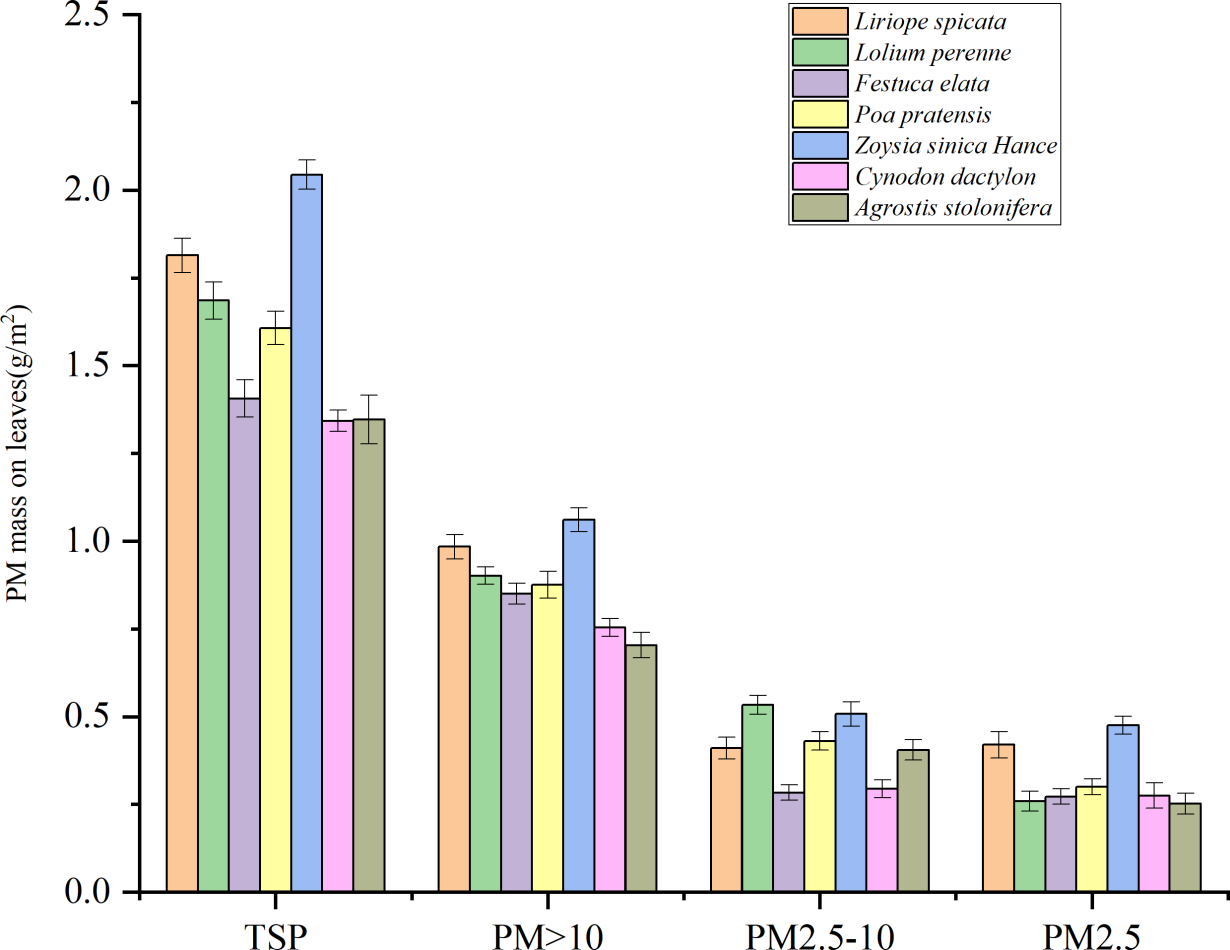
Maximum retention of PM of different particle sizes per unit leaf area of the experimental plants. Different lowercase letters indicate significant differences in the retention of PM of a particular size in different turf grasses (P < 0.05).

As shown in **Figure 4**, the results showed that the retention of particles of each size was significantly higher on the leaf surface of *Z. sinica* than on that of other plants (P < 0.05). The maximum TSP retention of the tested plants ranged from 1.34 to 2.04 g·m^-2^, and *A. stolonifera* and *C. dactylon* had the lowest maximum TSP retention. The maximum retention of PM_>10_ ranged from 0.70 to 1.06 g·m-2, and *A. stolonifera* had the lowest retention of PM_>10_. The maximum retention of PM_2.5-10_ ranged from 0.28 to 0.53 g·m^-2^, and *C. dactylon* and *F. elata* had the lowest retention of PM_2.5-10_. The maximum retention of PM_2.5_ ranged from 0.25 to 0.48 g·m^-2^, and *L. perenne* and *A. stolonifera* had the lowest retention of PM_2.5_.

### 3.2 Changes in the retention of particles of different sizes in turfgrass blades.

**Figure 5:**
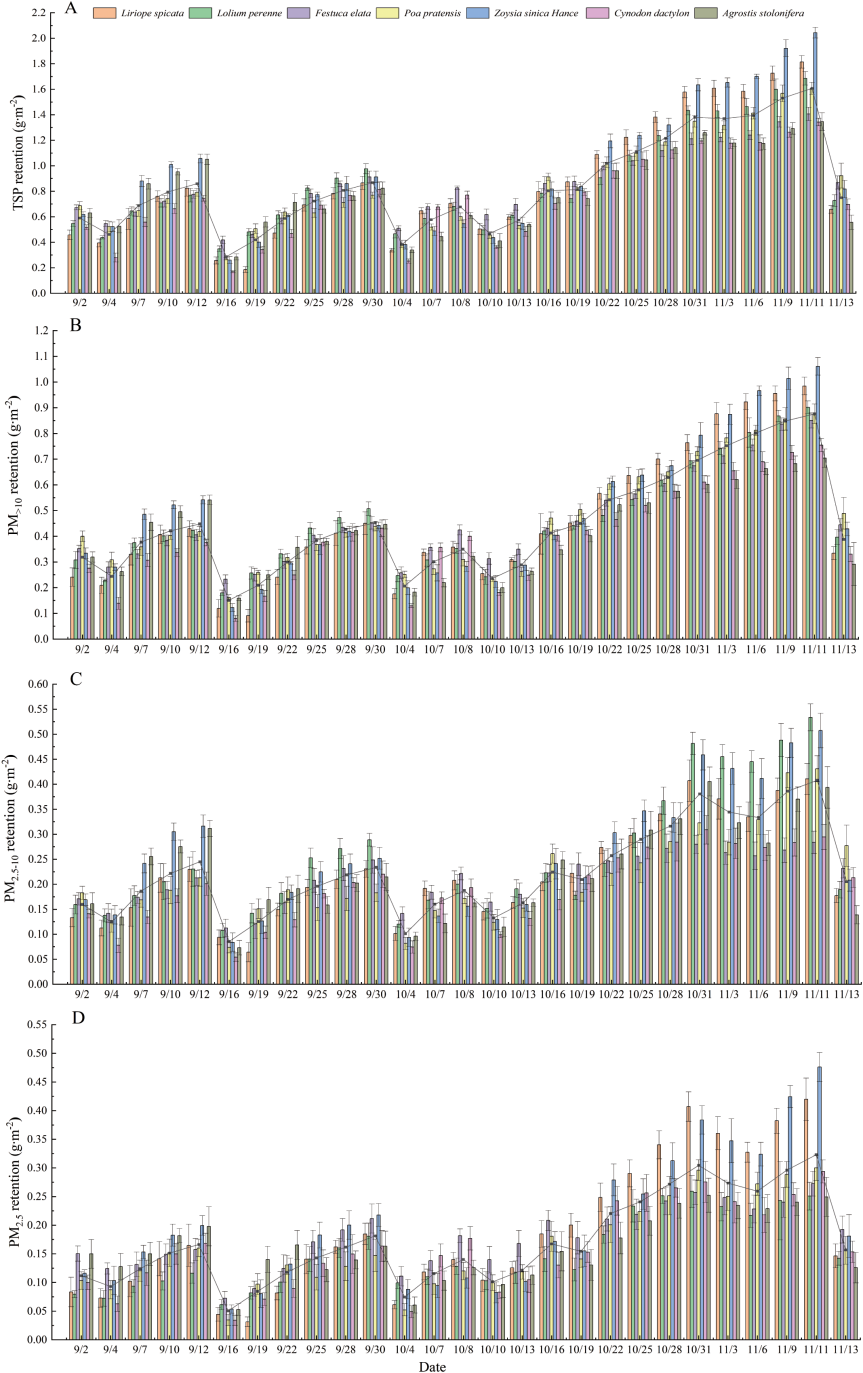
TSP, PM>10, PM2.5-10, and PM2.5 retention per unit leaf area of L. spicata, L. perenne, F. elata, P. pratensis, Z. sinica, C. dactylon, and A. stolonifera at each sampling event.

The retention of particles of different sizes on the leaf surface of the tested plants decreased significantly after rainfall (p<0.05) and then gradually increased. During the sampling period, the lowest value of PM appeared after 158 mm of rainfall, and the average retention of TSP was only 0.29 g·m-2. The values of TSP in the tested plants ranged from 0.17 to 2.04 g·m-2; the most variation in TSP retention was observed in *Z. sinica* (1.78 g·m^-2^), and the least variation in TSP retention was observed in *F. elata* (0.97 g·m^-2^) (**Figure 5-1**). The values of PM_>10_ for the tested plants ranged from 0.08 to 1.06 g·m-2; the most variation in PM_>10_ retention was observed in *Z. sinica* (0.94 g·m^-2^), and the least variation in PM_>10_ retention was observed for *A. stolonifera* (0.54 g·m^-2^) (**Figure 5-2**). Values of PM_2.5-10_ of the tested plants ranged from 0.05 to 0.53 g·m-2; the most variation in PM_2.5-10_ retention was observed in *L. perenne* (0.42g·m^-2^), which was 2.47 times higher than the variation in PM_2.5-10_ retention observed in *F. elata*, the species with the least variation in PM_2.5-10_ retention (**Figure 5-3**). The values of PM_2.5_ in the tested plants ranged from 0.03 to 0.48 g·m-2; the most variation in PM_2.5_ retention was observed in *Z. sinica* (0.43 g·m^-2^), which was 2.15 times higher than that observed in *L. perenne*, which was the species with the least variation in PM_2.5_ retention (**Figure 5-4**).

The overall dust retention of particles of different sizes of the seven types of lawn grass blades gradually increased over time; particles were displaced and replaced when rainfall and maximum wind speed were high. Rainfall had a significant effect on the removal of particles of different particle sizes in lawn grass blades, and the maximum wind speed had a weak effect on PM_>10_ and a stronger effect on PM_2.5-10_ and PM_2.5_.

### 3.3 Effect of leaf micromorphology on the retention of particles of different sizes

As shown in **Figures 1**, **2**, and **Table 2**, the groove proportion was significantly positively correlated with the retention of TSP and PM_>10_ (P<0.05) and highly significantly positively correlated with the retention of PM_2.5_ (P<0.01). There was no significant correlation between the groove width and particle retention on the leaf surface (P>0.01). Stomatal density was significantly positively correlated with TSP, PM_>10_, and PM_2.5_ retention (P<0.01). Stomatal size was negatively correlated only with PM_2.5_ retention (P<0.05). There was a significant negative correlation between leaf villous number and particle retention (P<0.05).

### 3.4 Effect of rainfall on the retention of particles of different sizes in lawn grass blades

**Figure 6:**
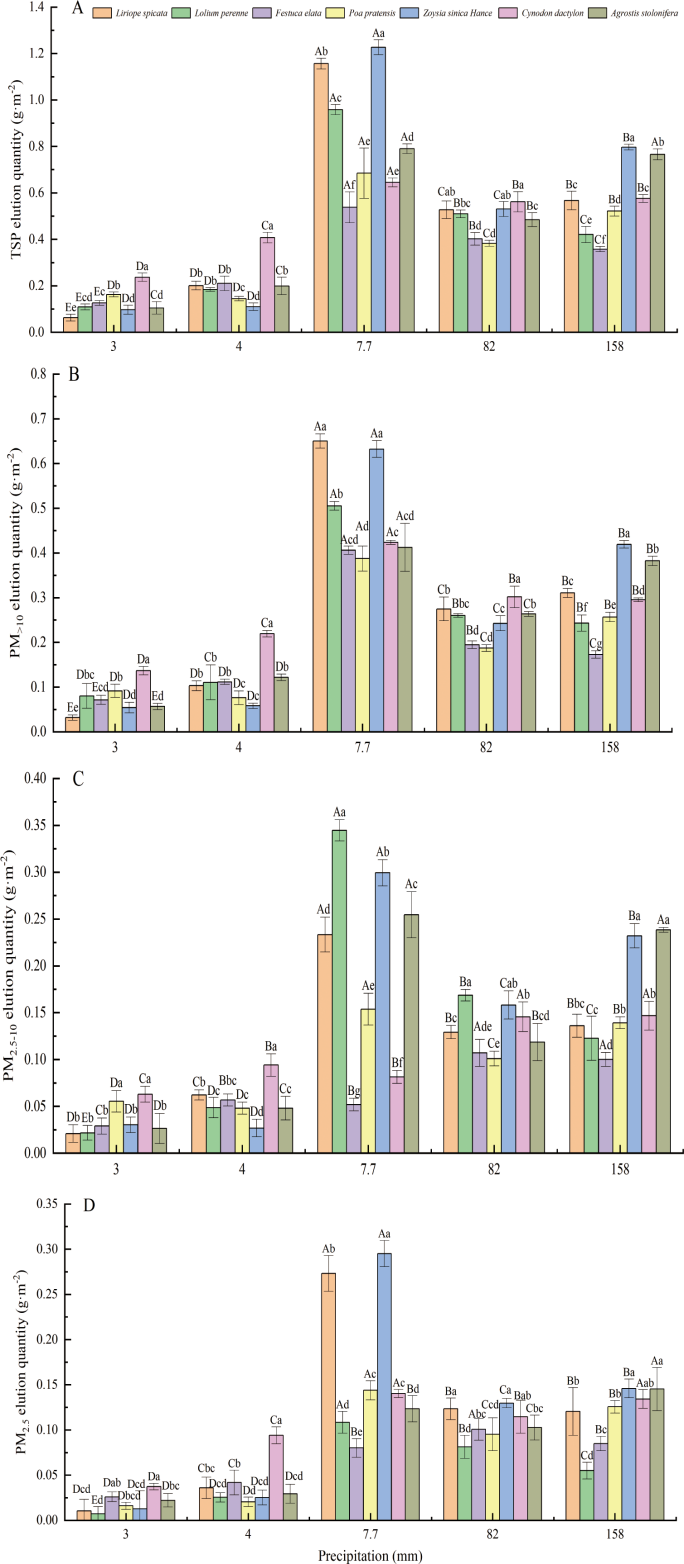
The amount of particles of different sizes eluted on the surface of seven types of turfgrass blades after rainfall. Different capital letters indicate significant differences in the retention of PM by the same plant after different amounts of rainfall; different lowercase letters indicate significant differences in the retention of PM by different plants after the same amount of rainfall.

Rainfall occurred on five days during the sampling period (**Figure 3**). After rainfall, the elution amount of PM_>10_ on the leaf surface of the tested plants was significantly higher than that of PM_2.5-10_ and PM_2.5_ (P<0.05), and no significant difference in the elution amount of PM_2.5-10_ and PM_2.5_ was observed (**Figure 6**). The elution of particles of different sizes on the leaf surface of the tested plants was highest after 7.7 mm of rainfall, followed by 82 and 158 mm of rainfall; particle elution was lowest after 3 and 4 mm of rainfall. However, there were some exceptions; for example, the elution of PM_2.5-10_ particles on the leaf surface of *F. elata* and *C. dactylon* was the highest after 82 and 152 mm of rainfall, followed by 4 and 7.7 mm of rainfall; the elution of PM_2.5-10_ particles on the leaf surface of these species was the lowest after 3 mm of rainfall.

The elution amount of particles of diferent sizes on the *C. dactylon* leaf surface was highest after 3 and 4 mm of rainfall (**Figure 6**). After 7 mm of rainfall, the elution amounts of TSP, PM_>10_, and PM_2.5_ on the leaf surface of *L. spicata* and *Z. sinica* were highest, and the elution amount of PM_2.5-10_ was highest for *L. perenne*. After 82 mm of rainfall, the elution amounts of TSP and PM_>10_ on the leaf surface of the tested plants were highest; the elution amount of PM_>10_ was highest in *C. dactylon*, the elution amount of PM_2.5-10_ was highest *L. perenne*, and the elution amount of PM_2.5_ was highest in *L. spicata* and *Z. sinica*. After 158 mm of rainfall, the highest amounts of PM were observed in *Z. sinica* and *A. stolonifera*. The total elution amount of each particle size on the leaf surface of the tested plants was highest in *Z. sinica*, followed by *L. spicata*, *C. dactylon*, *A. stolonifera*, *L. perenne*, *P. pratensis*, and *F. elata*.

### 3.5 Effect of maximum wind speed on the retention of particles of different sizes in lawn grass blades

**Figure 7:**
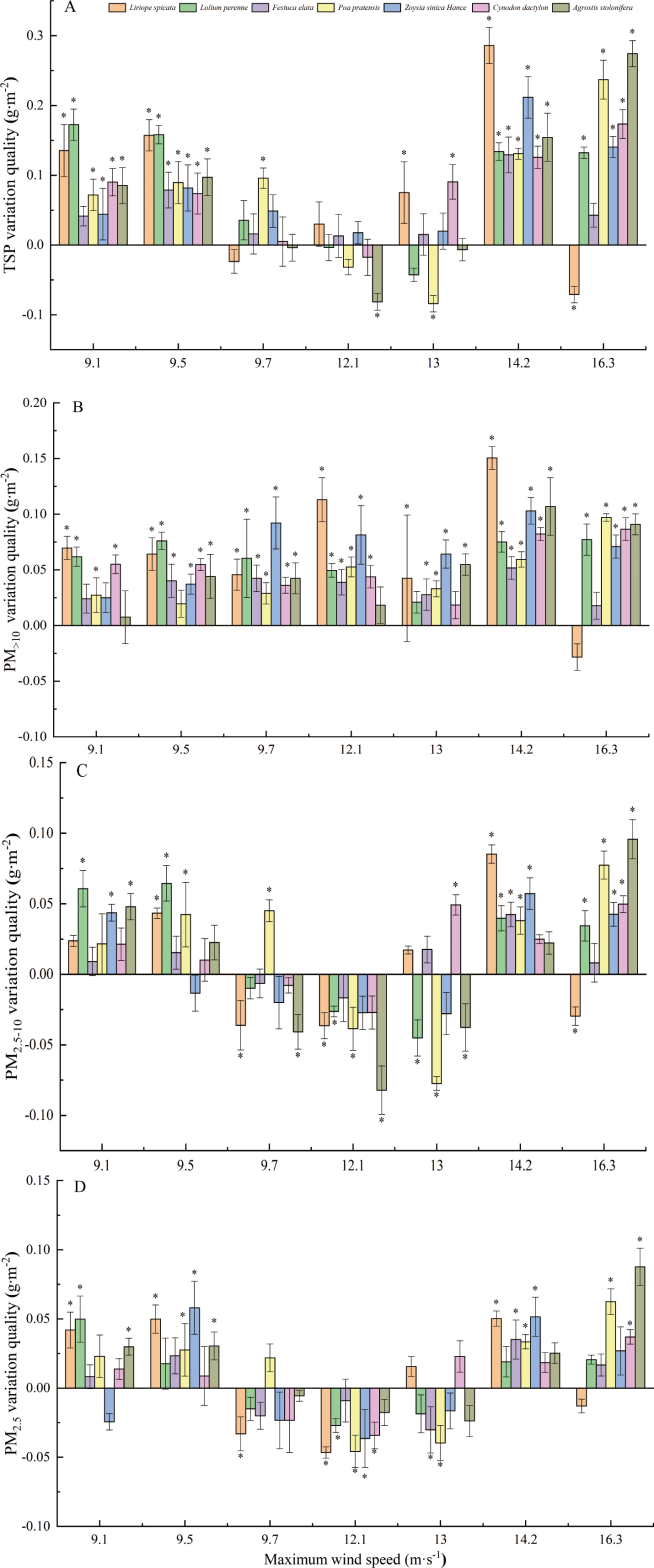
Variation in particles of different sizes on the surface of seven types of turfgrass blades after exposure to the maximum wind speed. (* indicates significant differences in the retention of particles on the leaves between two samples according to LSD tests).

The effect of maximum wind speed on the TSP retention per unit leaf area of tested plants is shown in **Figure 7-1**. The TSP retention of the tested plants significantly increased when the maximum wind speed was 9.1, 9.5 and 14.2 m·s^-1^. When the maximum wind speed was 12.1, 13, and 16.3 m·s-1, the retention of TSP on the leaf surface of *A. stolonifera*, *P. pratensis*, and *L. spicata* significantly decreased, respectively. Maximum wind speed did not significantly weaken the retention of PM_>10_ per unit leaf area of turfgrass (**Figure 7-2**). The retention of PM_>10_ in *L. spicata* leaves decreased by 0.03 g·m-2 after exposure to a maximum wind speed of 16.3 m·s-1, but this reduction was not significant (P >0.05). The effect of maximum wind speed on the retention of PM2.5-10 on the leaf surface of tested plants is shown in **Figure 7-3**. After exposure to maximum wind speeds of 9.1, 9.5, and 14.2 m·s-1, the retention of PM_2.5-10_ on the leaf surface of the tested plants increased significantly. Significant increases and decreases in the retention of PM_2.5-10_ on the leaf surface of the tested plants were observed following exposure to maximum wind speeds of 9.7, 13, and 16.3 m·s-1. After exposure to a maximum wind speed of 12.1 m·s-1, the retention of PM_2.5-10_ on the leaf surface of the tested plants was significantly reduced. The effect of maximum wind speed on the retention of PM_2.5_ per unit leaf area of lawn grasses is shown in **Figure 7-4**. The retention of PM_2.5_ significantly increased following exposure to the maximum wind speeds of 9.1, 9.5, 14.2, and 16.3 m·s-1. The retention of PM_2.5_ significantly decreased following exposure to maximum wind speeds of 9.7, 12.1, and 13 m·s^-1^.

Following exposure to maximum wind speeds, the reduction in particle retention on the middle surface of the tested plants was highest in *L. spicata*, followed by *A. stolonifera*, *Z. sinica*, *L. perenne*, *P. pratensis*, *C. dactylon*, and *F. elata*. When the grass blades contain a certain amount of dust, a maximum wind speed greater than 9.7 m·s-1 reduced the retention of TSP, PM_2.5-10_, and PM_2.5_ on the surface of the blades; maximum wind speed significantly promoted the retention of PM_>10_. In addition, when the dust retention of grass blades is low, a maximum wind speed of 16.3 m·s^-1^ did not reduce the dust retention of grass blades.

### 3.6 Effects of PM_10_ concentration, PM_2.5_ concentration, and relative humidity on the retention of particles of different sizes of lawn grass blades

**Figure 8:**
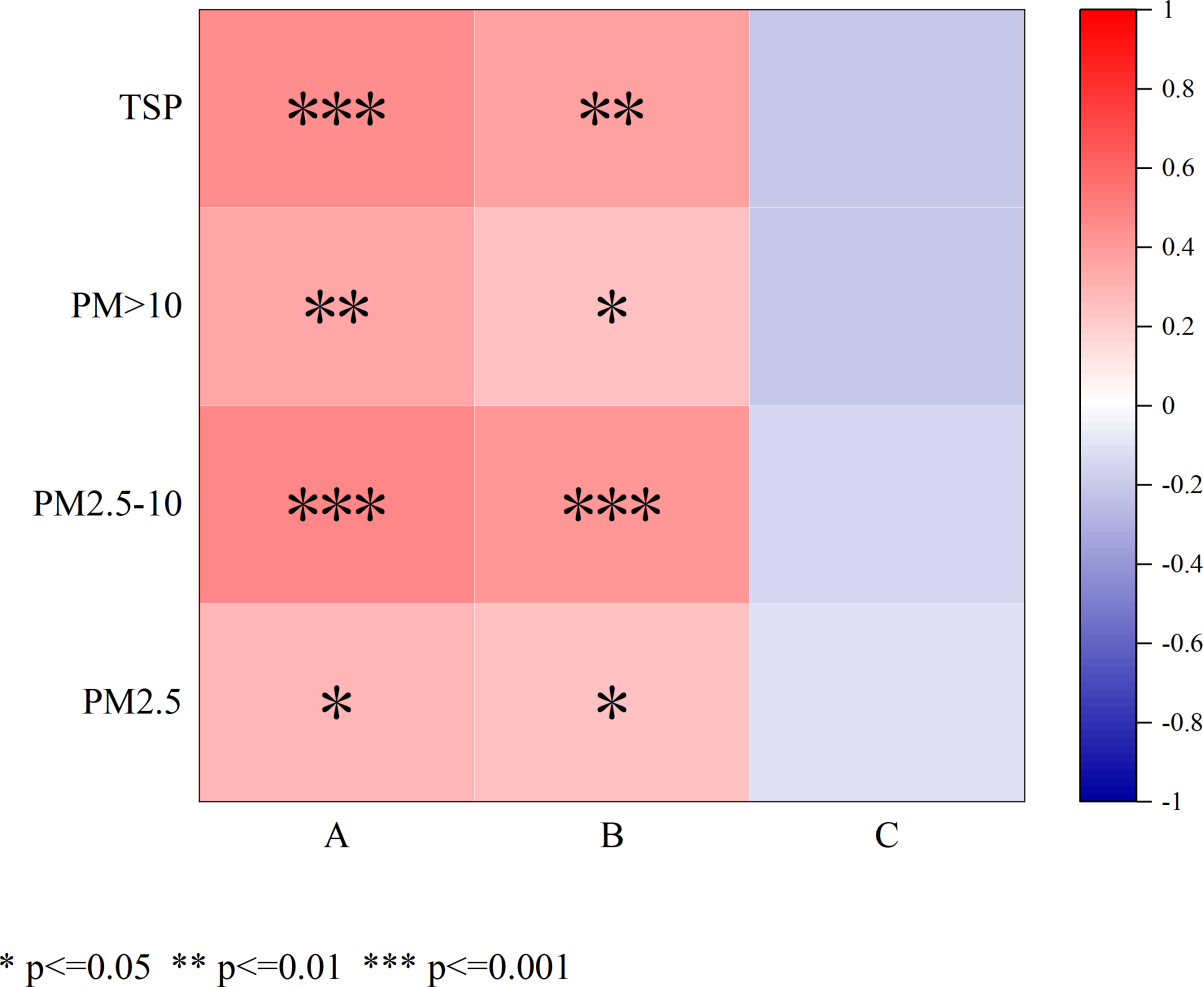
Relationship of PM10 concentration, PM2.5 concentration, and relative humidity with the retention of particles of different sizes on the blade surface. ”A” stands for “PM10 concentration,” “B” stands for “PM2.5 concentration,” and “C” stands for “Relative humidity.”.

The average daily PM_10_ concentration and average daily PM_2.5_ concentration were significantly positively correlated with the retention of particles of different sizes on the leaf surface of turfgrass (P<0.05) (**Figure 8**). There was no significant correlation between the average daily relative humidity and the retention of particles of different sizes on the leaf surface of turfgrass (P>0.05).

## 4 Discussion

### 4.1 Dust retention of the leaves of different plants and dynamic changes

The dust retention ability of the leaves of plants varies extensively among species and is affected by the roughness of leaves (Beckett et al., 1998), the shape and amount of lint (Rasanen et al., 2013), the groove proportion and groove width (Sabin et al., 2006), the content of wax (Saebo et al., 2012), and wettability and other factors (Leonard et al., 2016; Nowak et al., 2006; Rasanen et al., 2013; Speak et al., 2012). Sabin et al. (2006) found that the roughness of the leaf surface, the number of villi, and the content of mucous oil were positively correlated with the amount of dust retention on the leaf surface. Rasanen et al. (2013) also found that more particles could be retained in the foliage of plants with more villous, higher wettability, and low stomatal density. Kwak et al. (2020) concluded that stomatal size may be significantly related to the adsorption capacity of PM_2.5_ on the leaf surface, and stomatal density, capillary density, and roughness have weak effects on PM adsorption (Kwak et al., 2020). Li et al. suggested that a deeper surface texture of plant leaves was conducive to the capture of a greater number of particles. In addition, pore diameter length was significantly negatively correlated with PM retention, and pore density was significantly positively correlated with PM retention (Li et al., 2024).

The microstructure and characteristics of leaf surfaces play a pivotal role in the deposition and retention of PM. Leaf surfaces with dense villous and undulations significantly contribute to PM_2.5_ retention, as these structures provide increased surface area for particle adhesion through physical forces such as van der Waals forces or electrostatic interactions. The retention capacity is also influenced by the leaf’s roughness, with larger undulations enhancing the capture of PM_10_, suggesting that the physical texture of the leaf surface is crucial for PM retention (Prigioniero et al., 2023). Moreover, the specific variations in leaf microstructures can influence the phyllosphere microbial community by providing distinct microhabitats for PM-borne microorganisms. Dense leaf villous facilitate the capture of PM_2.5_ associated fungi, while bacteria are less impacted by PM and struggle to adhere to leaf microstructures (Xu et al., 2024).

The results of this study showed that the proportion of grooves and stomatal density were positively correlated with dust retention on the leaf surface, which was consistent with the results of previous studies. However, we found a negative correlation between the total amount of particles and particle retention, which is inconsistent with the results of previous studies. This can be explained by the fact that the leaves of Radix ophiopogonicum, which showed high dust retention in our study, lack a villous surface and a waxy layer. However, *A. stolonifera* and *C. dactylon* have a villous surface, but the dust retention of these surfaces is low. This indicates that the microstructural characteristics of the leaf surface affect the dust retention of the leaves, and the dust retention of the leaves is affected by various factors.

The dust retention of plants is affected by meteorological factors, such as precipitation, wind, and dust, and dust retention and dust fall occur simultaneously. Dust retention is thus a complex process. However, this study found that the overall dust retention still increased gradually, and some particles remained in the leaf surface of the seven plants tested after precipitation and maximum wind speed, indicating that precipitation and wind speed can only remove some particles, but their changes are different. Before rainfall, the retention of particles of each particle size increased with the increase of dust retention days. The variation of particle size after rainfall compared with that before rainfall showed a trend of TSP(0.17-2.04g ·m^-2^) >PM_>10_(0.08-1.06 g·m^-2^)>PM_2.5-10_(0.05-0.53g ·m^-2^) >PM_2.5_(0.03-0.48g ·m^-2^). This is consistent with the results of previous studies (Łukowski et al., 2020; Popek et al., 2013; Xu et al., 2022). In addition, precipitation can effectively impact TSP and PM_>10_, and PM_2.5-10_ and PM_2.5_ are more sensitive to wind speed. However, in this experiment, the maximum wind speed on September 19 and 22 did not reduce the amount of dust retention on the grass leaf surface of the lawn, possibly because the strong rainfall on September 14 and 15 had washed most of the particles on the leaf surface, and the maximum wind speed could no longer have a great impact on the variation of particles.

### 4.2 Effect of rainfall on leaf PM retention

The effect of rainfall on the retention of foliar particles varies; greater rainfall is generally thought to promote the elution of foliar particles (Yan et al., 2019; Zhou et al., 2021). In this study, the leaf PM elution amount was higher after 7.7 mm of rainfall than after 82 and 158 mm of rainfall, indicating that the leaf PM elution amount was not only affected by rainfall but also correlated with the accumulation of PM on the surface of plant leaves and the dust retention ability of plant leaves (Dzierzanowski et al., 2011; Popek et al., 2013). Species with rough leaf surfaces can capture more PM (Saebo et al., 2012), which in turn allows more PM to be washed away under the same amount of rainfall (Xu et al., 2017). In addition, 15 mm of precipitation is generally thought to be sufficient to wash all of the PM off the leaf surface, which initiates a new round of PM retention (Liu et al., 2013; Przybysz et al., 2014). In this study, even after a rainfall event of 158 mm, PM remained on the leaf surface of the tested plants, indicating that precipitation could not completely remove PM on the leaf surface (Przybysz et al., 2014; Xu et al., 2019).

We observed significant differences in the elution amounts of particles of different particle sizes on the leaf surface of tested plants under different amounts of rainfall; the cleaning effect of rainfall on particles on the leaf surface of different plants was closely related to the morphology of the leaf surface, including the grooves and villous (Chen et al., 2017). Leaf surfaces with densely ridged grooves can firmly hold a large number of particles (Wang et al., 2015). The leaves of *C. dactylon* leaves have many narrow grooves. During rainfall, raindrops first hit the leaves and splash the dust particles intercepted by the leaves; the raindrops then collect on the leaf surface and form runoff. In addition, the grooves are shallow, and the particles mostly rest on the leaf surface, which makes the particles easier to displace. This may be the reason why the particle elution rate on the leaf surface was higher under low amounts of rainfall; *F. elata* King has fewer and wider grooves with some microvillous. Grass leaves have villous and globular bulges, their grooves are not obvious, and their surfaces are rough. This may be the reason for the high particle elution rate on the leaf surface of *F. elata* and *Poa annua*, which is consistent with the results of Perini et al. indicating that leaf surface villous can reduce the strength of the scouring effect of rainfall events on leaf surface PM (Perini et al., 2017). Zhou et al. (2021) also found that the leaf surface dust retention capacity of P. annua was strong after rain. The amount of PM eluting on the *L. spicata* leaf surface was also high, and its leaf surface is smooth but villousless. Particles on plants with smooth leaf surfaces can be easily removed by rain (Beckett et al., 2010).

Previous studies suggest that precipitation can effectively wash out particles greater than 10 µm in size, and the ability of precipitation to displace PM_2.5-10_ and PM_2.5_ is poor (Przybysz et al., 2014; Wang et al., 2015; Xu et al., 2019; Zhou et al., 2020); however, these studies were all conducted on trees or shrubs. The effect of rainfall on the removal of PM from the leaf surface of herbs was stronger than on the removal of PM from the leaf surface of trees and shrubs (Zhou et al., 2021). Tomson et al. (2024) studied the removal effect of rainfall on PM on the leaf surface of green wall plants and found that rainfall had a significant scouring effect on small particles (PM_1_ and PM_10–2.5_). The scouring rate of large particles (PM_10–2.5_) was relatively low (Tomson et al., 2024). In our study, the elution amount of PM_>10_ was significantly greater than that of PM_2.5-10_ and PM_2.5_, but the elution amount of PM_2.5-10_ and PM_2.5_ also increased as the amount of rainfall increased, which was consistent with the results of Zhou et al. (2021). This might be related to two factors. First, the particles retained on the surface of lawn grass blades are mostly resting on the surface; they can thus be easily removed (Prusty et al., 2005; Wang et al., 2007). Second, turfgrass blades are mostly linear or lance-shaped, and there are several grooves in the blades; the raindrops form leaf surface runoff on the blade surface, which can mediate the removal of particles.

Based on the amount of dust retained on each type of lawn grass after rainfall, the content of retained PM of the seven lawn grasses was lowest after 82 mm and 158 mm of rainfall, and more PM remained on the leaf surface after 7.7 mm of rainfall; the difference between rainfall of 7.7 mm and 82 mm was substantial. The amount of rainfall that results in the optimal removal effect on the tested plants requires further study. In addition, the particle elution amount after 7.7 mm of rainfall was significantly higher than that after 82 mm and 158 mm of rainfall, indicating that the particle elution amount is not only related to rainfall but also to the content of retained particles on the leaf surface.

### 4.3 Effect of wind speed on leaf PM retention

The effect of different wind speeds on plant dust retention varies. Freer-Smith et al. (2004) found that when the wind speed increased from 3 m·s^-1^ to 9 m·s^-1^, the particle deposition rate increased, and the deposition rate of conifers was greater than that of deciduous trees. Beckett et al. suggested that when the wind speed is less than 8 m·s, the particle adsorption capacity increases as the wind speed increases, but continued increases in the wind speed may lead to a decrease in the particle adsorption capacity (Beckett et al., 2010). The relationship between the dust retention and wind speed of seven turfgrass species in this study was consistent with the findings of the above studies. When a certain amount of dust is present on the surface of turfgrass blades, a maximum wind speed of 9.1 m·s^-1^ can lead to a reduction in leaf PM retention; the leaf PM retention was not reduced, even if the maximum wind speed was 16.3 m·s^-1^. However, this leads to an increase in the retention of PM on the leaf surface.

Under high winds, the maximum reduction in total PM was 3.1 times higher in *L. spicata* than the lowest reduction in total PM observed in *F. elata*. Generally, the surface of the leaves is rough, the depth of the folds and grooves varies, and the amount of villous and waxy mucus on the surface is large; these all facilitate the interception and adsorption of PM and dust retention (Sgrigna et al., 2016; Weerakkody et al.,2018a). In contrast, leaves with a relatively smooth surface, a regular distribution of pores, and shallow and sparse grooves have a relatively weak dust retention ability (Weerakkody et al., 2018). In this study, the leaf surface of *L. spicata* is smooth, and the particles on the leaf surface can be easily displaced by wind; however, the leaf surface of *F. elata* has wide grooves, and the particles between the grooves contact the leaf surface and enter the villous; once attached to the villous, the particles are difficult to dislodge.

### 4.4 Effect of the PM_10_ concentration, PM_2.5_ concentration, and relative humidity on leaf PM retention

The concentration of PM in the air is correlated with the amount of dust retained on the leaves (Dang et al., 2022; Liu et al., 2013). Mori et al. (2015) found that the distribution of dust retained in highway shelterbelts was highly correlated with the PM_10_ concentration. Liu et al. found that the dust accumulation of leaves in industrial areas was higher than that in clean areas (Liu et al., 2013). Dang et al. (2022) also found that the retention of PM by plants is positively correlated with air quality, and the retention of TSP, PM_10_, and PM_2.5_ by plants is greater in industrial areas than in non-industrial areas. In this study, the direct effects of the PM10 concentration and PM_2.5_ concentration on the dust retention of seven turfgrass species were all positive, and changes in PM_10_ and PM_2.5_ concentrations were consistent with changes in PM_2.5-10_ concentrations, which was consistent with the results of previous studies.

The increase in relative humidity can condense particles in the air; at the same time, particles become larger by absorbing moisture in the air, which is conducive to particle sedimentation (Ruijgrok et al., 1997). During the study period, high relative humidity occurred mostly before and after rainfall, and the dust retention on the grass leaf surface was significantly affected by rainfall. Therefore, a clear correlation between relative humidity and dust retention on the grass leaf surface was not detected.

The relative humidity was high mostly a few days before or after rainfall. At this time, the effect of rainfall on the dust retention of the grass leaf surface was stronger, and a clear correlation between relative humidity and dust retention on the surface of grass leaves was not observed.

## 5 Conclusions

There were significant differences in the retention of TSP, PM_>10_, PM_2.5-10_, and PM_2.5_ on the blades of seven turfgrass species, and the dust retention ability of *Z. sinensis* was the strongest among all species tested. The dust retention on the leaf surface decreases after exposure to rainfall and wind, and this promotes the subsequent retention of dust particles. Weather factors had the strongest and weakest effect on the dust retention ability of *Z. sinensis* and *F. elata*, respectively. Rainfall can effectively elute particles of different sizes retained on lawn grass blades, and the amount of particles eluted increases as the amount of rainfall increases. In addition, the elution amount PM_>10_>PM_2.5-10_>PM_2.5_. When the leaf surface of the plants had a certain amount of dust, a maximum wind speed greater than 9.7 m·s^-1^ reduces the amount of dust retained on the leaves, and a maximum wind speed of 16.3 m ·s^-1^ does not reduce the amount of dust retained on grass leaves when the amount of dust retained on the grass blades is low. PM_10_ and PM_2.5_ concentrations in the environment were positively correlated with the retention of particles of different sizes on the leaf surface.In short, rainfall, maximum wind speed, and PM_10_ concentrations have significant effects on the ability of plant leaves to retain atmospheric particles. In practical applications, *Zoysia sinensis* and *Festuca elata* can be preferentially selected according to climatic conditions in different regions to reduce particulate matter in the air.The effects of these factors should be taken into account in studies of the accumulation of atmospheric particles on plant leaves.

## Data Availability

The raw data supporting the conclusions of this article will be made available by the authors, without undue reservation.

## References

[B1] Beckett K. P. Freer-Smith P. H. Taylor G. (1998). Urban woodlands: their role in reducing the effects of particulate pollution. Environ. Pollution. 99, 347–360. doi: 10.1016/S0269-7491(98)00016-5 15093299

[B2] Beckett K. P. Freer-Smith P. H. Taylor G. (2010). Particulate pollution capture by urban trees: effect of species and windspeed. Global Change Biol. 6, 995–1003. doi: 10.1046/j.1365-2486.2000.00376.x

[B3] Bui H. T. Odsuren U. Kwon K. J. Kim S. Y. Yang J. C. Jeong N. R.. (2021). Assessment of air pollution tolerance and particulate matter accumulation of 11 woody plant species. Atmosphere 12, 12. doi: 10.3390/atmos12081067

[B4] Burkhardt J. (2010). Hygroscopic particles on leaves: nutrients or desiccants? Ecol. Monogr. 80, 369–399. doi: 10.1890/09-1988.1

[B5] Chen G. J. Lin L. Hu Y. Zhang Y. X. Ma K. M. (2021). Net particulate matter removal ability and efficiency of ten plant species in Beijing. Urban. For. Urban. Green. 63, 8. doi: 10.1016/j.ufug.2021.127230

[B6] Chen L. X. Liu C. M. Zhang L. Zou R. Zhang Z. Q. (2017). Variation in tree species ability to capture and retain airborne fine particulate matter (PM2.5). Sci. Rep. 7, 11. doi: 10.1038/s41598-017-03360-1 28600533 PMC5466687

[B7] Chen Y. Wang X. Li M. Liu L. Xiang C. Li H.. (2024). Impact of trace elements on invasive plants: Attenuated competitiveness yet sustained dominance over native counterparts. Sci. Total. Environ. 927, 172292. doi: 10.1016/j.scitotenv.2024.172292 38588741

[B8] Chiam Z. Y. Song X. P. Lai H. R. Tan H. T. W. (2019). Particulate matter mitigation via plants: Understanding complex relationships with leaf traits. Sci. Total. Environment. 688, 398–408. doi: 10.1016/j.scitotenv.2019.06.263 31247483

[B9] Dang N. Zhang H. D. Salam M. M. A. Li H. M. Chen G. C. (2022). Foliar dust particle retention and metal accumulation of five garden tree species in Hangzhou: Seasonal changes. Environ. pollut. 306, 10. doi: 10.1016/j.envpol.2022.119472 35580713

[B10] Dzierzanowski K. Popek R. Gawronska H. Saebo A. Gawronski S. W. (2011). Deposition of particulate matter of different size fractions on leaf surfaces and in waxes of urban forest species. Int. J. Phytoremediation. 13, 1037–1046. doi: 10.1080/15226514.2011.552929 21972570

[B11] Escobedo F. J. Kroeger T. Wagner J. E. (2011). Urban forests and pollution mitigation: analyzing ecosystem services and disservices. Environ. pollut. 159, 2078–2087. doi: 10.1016/j.envpol.2011.01.010 21316130

[B12] Freer-Smith P. H. Beckett K. P. Taylor G. (2005). Deposition velocities to Sorbus aria, Acer campestre, Populus deltoides X trichocarpa ‘Beaupre’, Pinus nigra and X Cupressocyparis leylandii for coarse, fine and ultra-fine particles in the urban environment. Environ. Pollution. 133, 157–167. doi: 10.1016/j.envpol.2004.03.031 15327866

[B13] Freer-Smith P. H. El-Khatib A. A. Taylor G. (2004). Capture of particulate pollution by trees: A comparison of species typical of semi-arid areas (Ficus nitida and eucalyptus globulus) with european and north american species. Water. Air. Soil Pollution. 155, 173–187. doi: 10.1023/B:WATE.0000026521.99552.fd

[B14] Go T. Kim J. Lee S. J. (2021). Three-dimensional volumetric monitoring of settling particulate matters on a leaf using digital in-line holographic microscopy. J. Hazardous. Mater. 404, 7. doi: 10.1016/j.jhazmat.2020.124116 33049638

[B15] Guerrero-Leiva N. Castro S. A. Rubio M. A. Ortiz-Calderon C. (2016). Retention of atmospheric particulate by three woody ornamental species in Santiago, Chile. Water Air. Soil Pollution. 227, 9. doi: 10.1007/s11270-016-3124-4

[B16] Hofman J. Bartholomeus H. Calders K. Van Wittenberghe S. Wuyts K. Samson R. (2014). On the relation between tree crown morphology and particulate matter deposition on urban tree leaves: A ground-based LiDAR approach. Atmospheric. Environment. 99, 130–139. doi: 10.1016/j.atmosenv.2014.09.031

[B17] Huang M. Wang W. Leung H. Chan C. Y. Liu W. K. Wong M. H.. (2012). Mercury levels in road dust and household TSP/PM_2.5_ related to concentrations in hair in Guangzhou, China. Ecotoxicol. Environ. Saf. 81, 27–35. doi: 10.1016/j.ecoenv.2012.04.010 22579217

[B18] Hwang H.-J. Yook S.-J. Ahn K.-H. (2011). Experimental investigation of submicron and ultrafine soot particle removal by tree leaves. Atmospheric. Environment. 45, 6987–6994. doi: 10.1016/j.atmosenv.2011.09.019

[B19] Karottki D. G. Spilak M. Frederiksen M. Jovanovic Andersen Z. Madsen A. M. Ketzel M.. (2015). Indoor and outdoor exposure to ultrafine, fine and microbiologically derived particulate matter related to cardiovascular and respiratory effects in a panel of elderly urban citizens. Int. J. Environ. Res. Public Health 12, 1667–1686. doi: 10.3390/ijerph120201667 25648225 PMC4344687

[B20] Kwak M. J. Lee J. K. Park S. Kim H. Lim Y. J. Lee K.-A.. (2020). Surface-based analysis of leaf microstructures for adsorbing and retaining capability of airborne particulate matter in ten woody species. Forests 11 (9), 946–966. doi: 10.3390/f11090946

[B21] Kwon H. S. Ryu M. H. Carlsten C. (2020). Ultrafine particles: unique physicochemical properties relevant to health and disease. Exp. Mol. Med. 52, 318–328. doi: 10.1038/s12276-020-0405-1 32203103 PMC7156720

[B22] Leonard R. J. McArthur C. Hochuli D. F. (2016). Particulate matter deposition on roadside plants and the importance of leaf trait combinations. Urban. For. Urban. Green. 20, 249–253. doi: 10.1016/j.ufug.2016.09.008

[B23] Li G. Wang L. H. Sun F. B. Wang Y. J. Wu H. T. Hu Z. W.. (2020). Capacity of landscaping plants to accumulate airborne particulate matter in hangzhou, China. Pol. J. Environ. Stud. 29, 153–161. doi: 10.15244/pjoes/101606

[B24] Li Q. Liao J. Zhu Y. Ye Z. Chen C. Huang Y.. (2024). A study on the leaf retention capacity and mechanism of nine greening tree species in central tropical asia regarding various atmospheric particulate matter values. Atmosphere 15 (4), 394–407. doi: 10.3390/atmos15040394

[B25] Liang D. Ma C. Wang Y. Q. Wang Y. J. Zhao C. X. (2016). Quantifying PM2.5 capture capability of greening trees based on leaf factors analyzing. Environ. Sci. pollut. Res. 23, 21176–21186. doi: 10.1007/s11356-016-7687-9 PMC5099360 27646446

[B26] Liu L. Guan D. S. Peart M. R. Wang G. Zhang H. Li Z. W. (2013). The dust retention capacities of urban vegetation-a case study of Guangzhou, South China. Environ. Sci. pollut. Res. 20, 6601–6610. doi: 10.1007/s11356-013-1648-3 23608974

[B27] Łukowski A. Popek R. Karolewski P. (2020). Particulate matter on foliage of Betula pendula, Quercus robur, and Tilia cordata: deposition and ecophysiology. Environ. Sci. pollut. Res. 27, 10296–10307. doi: 10.1007/s11356-020-07672-0 PMC7118030 31933074

[B28] Marien B. Marien J. Xuan H. N. The C. N. Van S. N. Samson R. (2019). Particulate matter accumulation capacity of plants in Hanoi, Vietnam. Environ. Pollution. 253, 1079–1088. doi: 10.1016/j.envpol.2019.07.035 31434185

[B29] Mori J. Hanslin H. M. Burchi G. Saebo A. (2015). Particulate matter and element accumulation on coniferous trees at different distances from a highway. Urban. Forestry. Urban. Greening. 14, 170–177. doi: 10.1016/j.ufug.2014.09.005

[B30] Nowak D. J. Crane D. E. Stevens J. C. (2006). Air pollution removal by urban trees and shrubs in the United States. Urban. For. Urban. Green. 4, 115–123. doi: 10.1016/j.ufug.2006.01.007

[B31] Nowak D. J. Hirabayashi S. Doyle M. McGovern M. Pasher J. (2018). Air pollution removal by urban forests in Canada and its effect on air quality and human health. Urban. Forestry. Urban. Greening. 29, 40–48. doi: 10.1016/j.ufug.2017.10.019

[B32] Ould-Dada Z. Baghini N. M. (2001). Resuspension of small particles from tree surfaces. Atmospheric. Environment. 35, 3799–3809. doi: 10.1016/S1352-2310(01)00161-3

[B33] Perini K. Ottele M. Giulini S. Magliocco A. Roccotiello E. (2017). Quantification of fine dust deposition on different plant species in a vertical greening system. Ecol. Eng. 100, 268–276. doi: 10.1016/j.ecoleng.2016.12.032

[B34] Popek R. Gawronska H. Wrochna M. Gawronski S. W. Saebo A. (2013). Particulate matter on foliage of 13 woody species: deposition on surfaces and phytostabilisation in waxes – a 3-year study. Int. J. Phytoremediation. 15, 245–256. doi: 10.1080/15226514.2012.694498 23488010

[B35] Popek R. Przybysz A. Gawronska H. Klamkowski K. Gawronski S. W. (2018). Impact of particulate matter accumulation on the photosynthetic apparatus of roadside woody plants growing in the urban conditions. Ecotox. Environ. Safe. 163, 56–62. doi: 10.1016/j.ecoenv.2018.07.051 30036757

[B36] Prigioniero A. Postiglione A. Zuzolo D. Niinemets Ü. Tartaglia M. Scarano P.. (2023). Leaf surface functional traits influence particulate matter and polycyclic aromatic hydrocarbons air pollution mitigation: Insights from Mediterranean urban forests. J. Cleaner. Production. 418, 138158. doi: 10.1016/j.jclepro.2023.138158

[B37] Prusty B. A. Mishra P. C. Azeez P. A. (2005). Dust accumulation and leaf pigment content in vegetation near the national highway at Sambalpur, Orissa, India. Ecotoxicol. Environ. Saf. 60, 228–235. doi: 10.1016/j.ecoenv.2003.12.013 15546639

[B38] Przybysz A. Saebo A. Hanslin H. M. Gawronski S. W. (2014). Accumulation of particulate matter and trace elements on vegetation as affected by pollution level, rainfall and the passage of time. Sci. Total. Environ. 481, 360–369. doi: 10.1016/j.scitotenv.2014.02.072 24607629

[B39] Qin H. Q. Hong B. Jiang R. S. Yan S. S. Zhou Y. H. (2019). The effect of vegetation enhancement on particulate pollution reduction: CFD simulations in an urban park. Forests 10, 22. doi: 10.3390/f10050373

[B40] Qin Y. Zhang H. Liu Q. Y. Jiang B. Chen J. J. Zhang T. (2021). Sulforaphane attenuates oxidative stress and inflammation induced by fine particulate matter in human bronchial epithelial cells. J. Funct. Foods. 81, 9. doi: 10.1016/j.jff.2021.104460

[B41] Rasanen J. V. Holopainen T. Joutsensaari J. Ndam C. Pasanen P. Rinnan A.. (2013). Effects of species-specific leaf characteristics and reduced water availability on fine particle capture efficiency of trees. Environ. Pollution. 183, 64–70. doi: 10.1016/j.envpol.2013.05.015 23735814

[B42] Rodriguez-Germade I. Mohamed K. J. Rey D. Rubio B. Garcia A. (2014). The influence of weather and climate on the reliability of magnetic properties of tree leaves as proxies for air pollution monitoring. Sci. Total. Environ. 468-469, 892–902. doi: 10.1016/j.scitotenv.2013.09.009 24080416

[B43] Ruijgrok W. Tieben H. Eisinga P. (1997). The dry deposition of particles to a forest canopy: A comparison of model and experimental results. Atmospheric. Environment. 31, 399–415. doi: 10.1016/S1352-2310(96)00089-1

[B44] Sabin L. Heelim J. Teresavenezia M. Winer A. Schiff K. Stolzenbach K. (2006). Dry deposition and resuspension of particle-associated metals near a freeway in Los Angeles. Atmospheric. Environment. 40, 7528–7538. doi: 10.1016/j.atmosenv.2006.07.004

[B45] Saebo A. Popek R. Nawrot B. Hanslin H. M. Gawronska H. Gawronski S. W. (2012). Plant species differences in particulate matter accumulation on leaf surfaces. Sci. Total. Environ. 427, 347–354. doi: 10.1016/j.scitotenv.2012.03.084 22554531

[B46] Sgrigna G. Baldacchini C. Dreveck S. Cheng Z. Calfapietra C. (2020). Relationships between air particulate matter capture efficiency and leaf traits in twelve tree species from an Italian urban-industrial environment. Sci. Total. Environment. 718, 12. doi: 10.1016/j.scitotenv.2020.137310 32088481

[B47] Sgrigna G. Baldacchini C. Esposito R. Calandrelli R. Tiwary A. Calfapietra C. (2016). Characterization of leaf-level particulate matter for an industrial city using electron microscopy and X-ray microanalysis. Sci. Total. Environment. 548, 91–99. doi: 10.1016/j.scitotenv.2016.01.057 26802337

[B48] Speak A. F. Rothwell J. J. Lindley S. J. Smith C. L. (2012). Urban particulate pollution reduction by four species of green roof vegetation in a UK city. Atmos. Environ. 61, 283–293. doi: 10.1016/j.atmosenv.2012.07.043

[B49] Steubing L. (1982). Problems of bioindication and the necessity of standardization.In Monitoring of air pollutants by plants-Methods and problems. Der. Internist. 884-885, 49–52.

[B50] Sun X. D. Li H. M. Guo X. Sun Y. K. Li S. M. (2018). Capacity of six shrub species to retain atmospheric particulates with different diameters. Environ. Sci. pollut. Res. 25, 2643–2650. doi: 10.1007/s11356-017-0549-2 29134522

[B51] Tiwary A. Morvan H. P. Colls J. J. (2006). Modelling the size-dependent collection efficiency of hedgerows for ambient aerosols. J. Aerosol. Sci. 37, 990–1015. doi: 10.1016/j.jaerosci.2005.07.004

[B52] Tomson M. Kumar P. Abhijith K. V. Watts J. F. (2024). Exploring the interplay between particulate matter capture, wash-off, and leaf traits in green wall species. Sci. Total. Environ. 921, 170950. doi: 10.1016/j.scitotenv.2024.170950 38360301

[B53] Vos P. E. J. Maiheu B. Vankerkom J. Janssen S. (2013). Improving local air quality in cities: To tree or not to tree? Environ. Pollut. 183, 113–122. doi: 10.1016/j.envpol.2012.10.021 23194646

[B54] Wang H. Shi H. Wang Y. (2015). Effects of weather, time, and pollution level on the amount of particulate matter deposited on leaves of Ligustrum lucidum. ScientificWorldJournal 2015, 935942. doi: 10.1155/2015/935942 25685849 PMC4313054

[B55] Wang H. Zhang B. Ni Y. Kuti J. L. Chen B. Chen M.. (2007). Pharmacodynamic target attainment of seven antimicrobials against Gram-negative bacteria collected from China in 2003 and 2004. Int. J. Antimicrob. Agents. 30, 452–457. doi: 10.1016/j.ijantimicag.2007.06.005 17646088

[B56] Weerakkody U. Dover J. W. Mitchell P. Reiling K. (2018a). Evaluating the impact of individual leaf traits on atmospheric particulate matter accumulation using natural and synthetic leaves. Urban. Forestry. Urban. Greening. 30, 98–107. doi: 10.1016/j.ufug.2018.01.001

[B57] Weerakkody U. Dover J. W. Mitchell P. Reiling K. (2018b). The impact of rainfall in remobilising particulate matter accumulated on leaves of four evergreen species grown on a green screen and a living wall. Urban. For. Urban. Green. 35, 21–31. doi: 10.1016/j.ufug.2018.07.018

[B58] Xu L. Liu Y. Feng S. Liu C. Zhong X. Ren Y.. (2024). The relationship between atmospheric particulate matter, leaf surface microstructure, and the phyllosphere microbial diversity of Ulmus L. BMC Plant Biol. 24, 566. doi: 10.1186/s12870-024-05232-z 38880875 PMC11181616

[B59] Xu L. S. Yan Q. Liu L. W. He P. Zhen Z. L. Duan Y. H.. (2022). Variations of particulate matter retention by foliage after wind and rain disturbance. Air Qual. Atmos. Health. 15, 437–447. doi: 10.1007/s11869-021-01086-8

[B60] Xu Y. S. Xu W. Mo L. Heal M. R. Xu X. W. Yu X. X. (2018). Quantifying particulate matter accumulated on leaves by 17 species of urban trees in Beijing, China. Environ. Sci. pollut. Res. 25, 12545–12556. doi: 10.1007/s11356-018-1478-4 29464604

[B61] Xu X. W. Yu X. X. Bao L. Desai A. R. (2019). Size distribution of particulate matter in runoff from different leaf surfaces during controlled rainfall processes. Environ. pollut. 255, 8. doi: 10.1016/j.envpol.2019.113234 31541810

[B62] Xu X. W. Zhang Z. M. Bao L. Mo L. Yu X. X. Fan D. X.. (2017). Influence of rainfall duration and intensity on particulate matter removal from plant leaves. Sci. Total. Environ. 609, 11–16. doi: 10.1016/j.scitotenv.2017.07.141 28732292

[B63] Yan G. X. Cong L. Zhai J. X. Wu Y. N. Dai L. Y. Zhang Z. M. (2019). Particle removal in polluted cities: Insights from the wash-off process dynamics for different wetland plants. J. Environ. Manage. 245, 114–121. doi: 10.1016/j.jenvman.2019.05.085 31150902

[B64] Zhang W. Y. Zhang Y. Z. Gong J. R. Yang B. Zhang Z. H. Wang B.. (2020). Comparison of the suitability of plant species for greenbelt construction based on particulate matter capture capacity, air pollution tolerance index, and antioxidant system. Environ. pollut. 263, 12. doi: 10.1016/j.envpol.2020.114615

[B65] Zhou S. J. Cong L. Liu Y. Xie L. M. Zhao S. Q. Zhang Z. M. (2021). Rainfall intensity plays an important role in the removal of PM from the leaf surfaces. Ecol. Indic. 128, 9. doi: 10.1016/j.ecolind.2021.107778

[B66] Zhou S. J. Yan G. X. Wu Y. N. Zhai J. X. Cong L. Zhang Z. M. (2020). The PM removal process of wetland plant leaves with different rainfall intensities and duration. J. Environ. Manage. 275, 9. doi: 10.1016/j.jenvman.2020.111239 32846360

